# Diagnosis of multiple tuberculous muscle abscesses in a patient with systemic lupus erythematosus by metagenomic next-generation sequencing- a case report and literature review

**DOI:** 10.1186/s12879-024-09179-2

**Published:** 2024-03-04

**Authors:** Liu Wentao, Xie Shuxia, Zhu Guoxing, Chen Qiaoping, Chen Peiran, Wu Angela, Li Meirong, Yin Songchao, Feng Peiying

**Affiliations:** 1 Department of Dermatology, 3rd Affiliated Hospital, Sun Yat-Sen University, Guangzhou, China; 2 Department of Allergy, 3rd Affiliated Hospital, Sun Yat-Sen University, Guangzhou, China

**Keywords:** Extremity, Metagenomic next-generation sequencing technology, Tuberculous muscle abscess, *Mycobacterium tuberculosis*, Systemic lupus erythematosus

## Abstract

**Background:**

Early diagnosis of muscular tuberculosis (TB) without coexistent active skeletal involvement is often challenging because the disease is very rare and its clinical manifestation is nonspecific and misleading. To raise the awareness and emphasize early diagnosis of muscular TB, we present a case of multiple tuberculous muscle abscesses in a systemic lupus erythematosus (SLE) female, but without pulmonary tuberculosis (PTB), in order to increase awareness of and stress the need of early detection of muscular TB.

**Case presentation:**

A 44-year-old woman with a 6-year history of SLE who had been treated with methylprednisolone for a long time complained of erythema on her trunk and extremities for five months, along with edema and myalgia for two months, and fever for one month. The patient was first misdiagnosed as SLE overlap dermatomyositis. However, an ultrasound-guided drainage of muscle abscesses revealed positive acid-fast staining combined with positive deoxyribonucleic acid fragment of *Mycobacterium tuberculosis* using metagenomic next-generation sequencing (mNGS). The patient was cured and released following standard anti-tuberculosis medication, local puncture drainage, and an intravitreal injection of streptomycin. Literature search found only 19 cases of tuberculous muscle abscesses occurring in the extremities reported from 1999 to 2023.

**Conclusions:**

Extrapulmonary TB with predominantly muscle involvement is rare and with no specific clinical presentation. Muscular tuberculosis may be disdiagnosed for dermatomyositis due to the high muscle enzyme levels, delaying diagnosis and treatment. mNGS technology is helpful in the early and rapid diagnosis of muscular TB. On the basis of traditional anti-tuberculosis treatment, an ultrasound-guided percutaneous puncture drainage and intracavitary injection of streptomycin for the treatment of tuberculous muscle abscess is easy to operate, safe and effective, which is worthy of clinical popularization and application.

## Background

Tuberculosis (TB) is a chronic infectious disease caused by *Mycobacterium tuberculosis* infection, which could involve multiple organs and present as pathological caseous granulomas. It is highly contagious and affects the entire population, ranking as the second leading infectious killer after COVID-19 in 2021, in which a total of 1.6 million people died from TB [1]. Tuberculosis can be classified into PTB and extrapulmonary TB based on the site of infection. The latter mainly includes musculoskeletal tuberculosis, lymph node tuberculosis, pleural tuberculosis, and visceral tuberculosis. However, muscular TB is relatively rare. It was reported that tuberculous myositis accounted for approximately 0.0026%of all TB cases abroad, and a prevalence of 1.8% in Asia [2–4]. There are a limited number of reports that describe tuberculous muscle abscesses occurring in the extremities. Herein we presented a case of multiple tuberculous muscle abscesses in a systemic lupus erythematosus (SLE) female, but without PTB. In addition, the clinical characteristics, laboratory testing, diagnosis, and treatment methods of tuberculous muscle abscesses were discussed, aiming to deepen our understanding of extrapulmonary TB.

## Case presentation

A 44-year-old woman was admitted to the Third Affiliated Hospital of Sun Yat-sen University in November 2022 because of erythema of the trunk and extremities for five months, edema and myalgia for two months, and fever for one month. The patient had been diagnosed as SLE in another hospital for six years ago due to malar rash, alopecia, and positive autoantibodies including ANA (cytoplasmic type), anti-dsDNA, and anti-Sm antibodies. She was treated with methylprednisolone 32 mg/d and hydroxychloroquine 200 mg/d, and the condition was gradually controlled. Low dose methylprednisolone (8 mg/d) has been administered consistently since June 2022. Five months before hospitalization, she had local erythema on her chest, back, and upper limbs without pain and itching. The patient was suspected of overlap syndrome of SLE with dermatomyositis in another hospital, as autoantibodies including anti-nRNP, anti-SSA, anti-Ro 52, anti-histones, and anti-PL-7 antibodies were positive, and peripheral blood myocardial enzyme spectrum showed elevated serum creatine kinase (CK) 25,170 U/L, creatine kinase isoenzyme (CK-MB) 253 U/L and lactate dehydrogenase (LDH) 1538 U/L. There was no improvement after treatment with cyclophosphamide (0.6-0.8 g/w), methylprednisolone (36 mg/d) and hydroxychloroquine (100 mg/d). The patient showed observable swellings and myalgia at the erythema sites three months later, although there was also a significant decrease in blood CK (749 U/L) and LDH (463 U/L). But the patient’s health worsened, exhibiting chills and a high fever that ranges from 39 to 40 degrees Celsius. Color Doppler ultrasound imaging of the swelling left thigh showed diffuse hypoechoic areas in the deep muscle layer, suggesting skin and soft tissue infection. Combinations of vancomycin 1 mg/kg plus imipenem 5 mg/kg were used for the treatment, but the patient’s condition worsened and transferred to our hospital for further treatment. At admission, the patient had obvious malaise, drowsiness and night sweats.

**Fig. 1:**
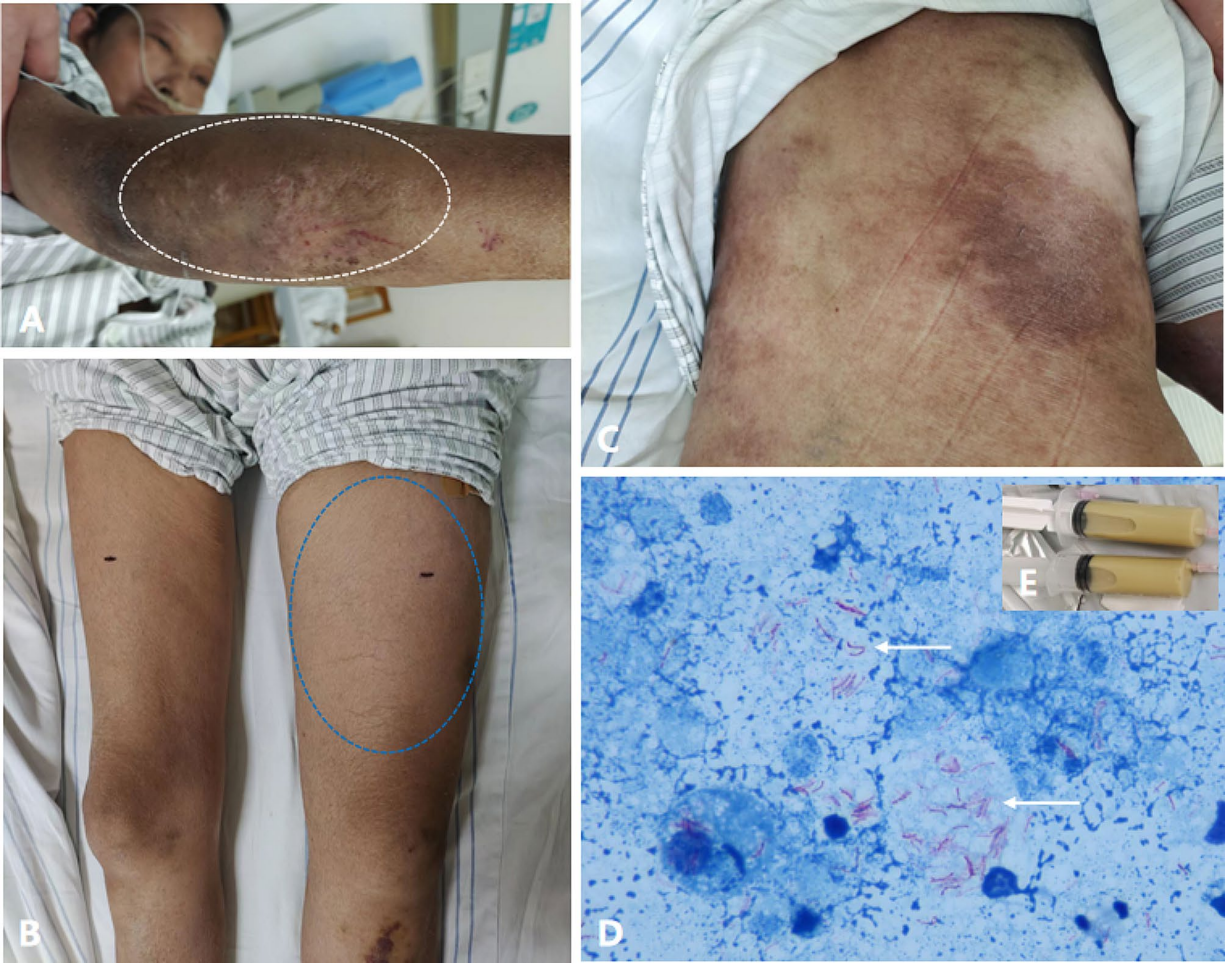
Localization of soft tissue swellings on the front of right ulna (**A**) and left femur (**B**). The sites of abscesses were defined by white circle and blue circle, respectively. Hyperpigmented macules and patches distributed on the back (**C**). Abscess pus drainage from the left thigh and limb (**E**) revealed positive staining for acid fast bacilli (**D**) (white arrows). (Ziehl-Neelsen stain, × 400)

A physical examination revealed a temperature of 38.4 °C, a pulse rate of 90 beats per minute, a blood pressure of 121/83 mmHg, and a respiratory rate of 20 beats per minute. There were diffusely soft tissue swellings on the right lower upper arm, the front of right ulna and the left femur. With rash and pain, the temperature of the skin overlying the affected area is marginally higher (Fig. 1A&B). The patient presented with hyperpigmented macules and patches over the face, neck, upper limbs, chest and back (Fig. 1C). The upper and lower limbs were normal, whereas the lower left limb’s manual muscle testing was rated at 2/5.

**Fig. 2:**
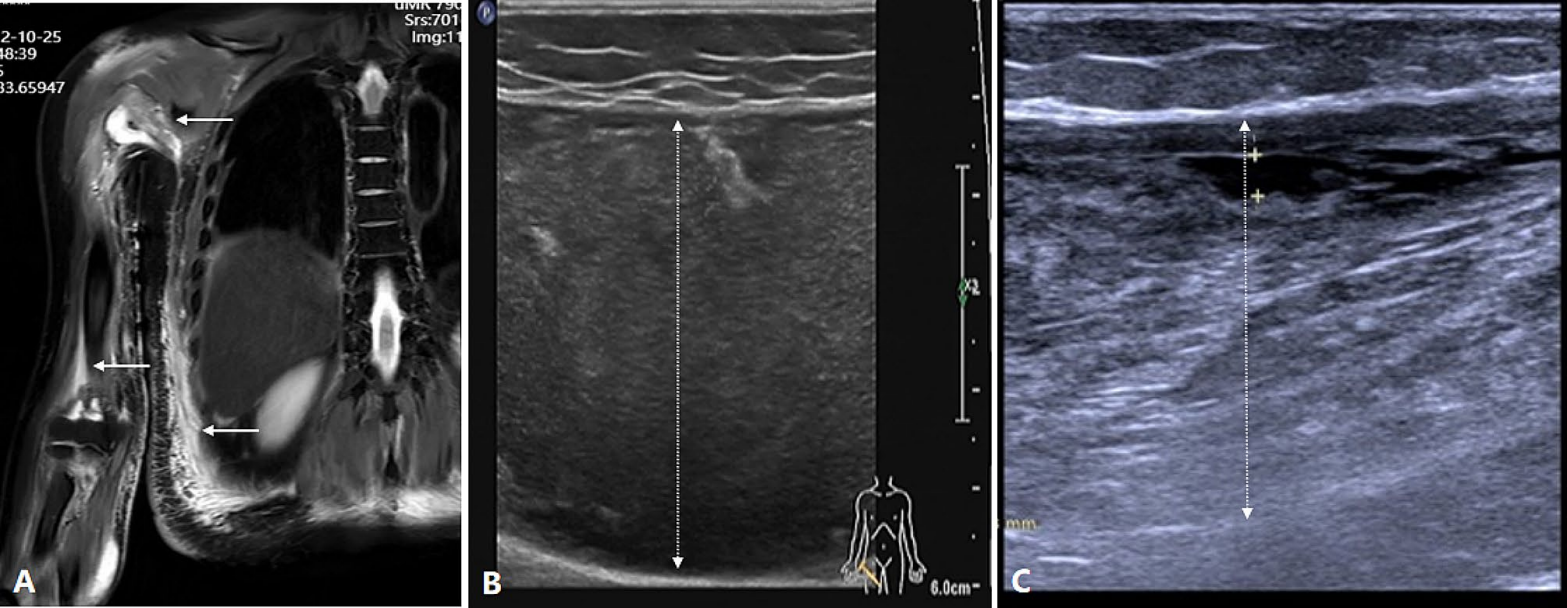
MRI of subcutaneous abscesses in the upper right limb and waist (**A**). The sites of involved muscle with high signals were defined by white arrows. Ultrasound revealed signs of inflammation and local fluid in left thigh (**B**), and the abscess faded obviously after treatment for 2 months (**C**)

Laboratory tests showed white blood cells 6.34 × 109/L, and the percentage of neutrophil was 92.7%. Serum CK was 25 U/L and LDH 201 U/L, C3 was decreased at 0.64 g/L (0.9–1.8 g/L) but C4 was normal at 0.2 g/L (0.1–0.4 g/L). Her erythrocyte sedimentation rate was elevated at 9 mm/hour (< 20 mm/hour) and C-reactive protein mildly elevated at 5.2 mg/L (< 5 mg/L). The results of T-cell spot of tuberculosis assay (T-SPOT) and tuberculin PPD skin test were positive. Computed tomography (CT) scans did not find any active TB lesion in the lung. Ultrasound revealed signs of inflammation and local fluid in the right arm and left thigh (Fig. 2B). Magnetic resonance imaging (MRI) showed inflammatory changes along the right upper arm, ulna, back and waist (Fig. 2A) and the left femur, with different fluid collection extension along the path of subcutaneous connective tissue. Large volumes of pus were drained from the abscess on the left thigh and right upper leg using ultrasound-guided fine-needle aspiration (Fig. 1E), and the pus tested strongly positive for Ziehl-Neelsen staining (4+) (Fig. 1D). An ultrasound-guided drainage of muscle abscesses revealed positive acid-fast staining, The metagenomic next-generation sequencing (mNGS) revealed 266,624 total reads of *M. tuberculosis* complex with 100% relative abundance. Once upon confirming the tuberculous muscle infection, anti-TB regimen was initiated, including isoniazid (300 mg qd), moxifloxacin (400 mg qd), ethambutol (300 mg qd), pyrazinamide (300 mg qd). In the meantime, 1.0 g of streptomycin was directly injected into the left thigh abscess after roughly 300 ml of yellow pus were removed per puncture during twice-weekly percutaneous pus drainage. No complications occurred during the process of puncture, drainage and drug injection. The patient’s general condition improved significantly within the first week, and the abscesses faded obviously after 2 months of treatment (Fig. 2C). The patient was discharged and accepted postoperative anti-TB medication for 12 months. At one-year follow-up, ultrasound did not find any abscess recurrence.

## Discussion and conclusions

The WHO Global Tuberculosis Report 2022 provides that there has been an increasing trend in the number of TB cases, drug-resistant TB cases, and tuberculosis-related deaths worldwide in recent years, indicating that many TB patients are still not receiving proper diagnosis and treatment [1]. This disease can be transmitted through the air and primarily affects the lungs, accounting for 80-85% of all TB cases. It can also spread through the bloodstream or lymphatic system to organs and tissues outside the lungs, resulting in extrapulmonary TB. The symptoms of extrapulmonary TB vary depending on the site of infection, with lymph node TB and tuberculous meningitis being the most common. A nationwide cross-sectional study conducted in China from 2020 to 2021, which involved 6843 TB cases, revealed that extrapulmonary TB accounted for 24.6% of all cases, with isolated extrapulmonary TB accounting for 21.3%. Among the cases of extrapulmonary TB, 15.8% of the extrapulmonary tuberculosis patients involved the musculoskeletal system; these cases included limb bone TB (0.5%), muscle TB (1.0%), spinal TB (9.8%), and other bone TB (2.0%) [5]. A retrospective study conducted in Taiwan from 1996 to 2001 showed that only 1.8% (21/1153) of tuberculosis cases belong to TB myositis [4]. Consequently, it is evident that tuberculous myositis and TB infections in the musculoskeletal system are uncommon, which increases the likelihood of missed and incorrect diagnoses.

The patient in this case had underlying SLE and multiple tuberculous muscle abscesses in the trunk and limbs. To the best of our knowledge, 20 cases in which TB was associated with tuberculous abscesses in the limbs and back muscle have been reported in the literature since 1999 (Table 1), including our case accounting for approximately 6% (22/365) of muscle system TB infections [6–21]. This entity often has an insidious onset and is characterized by fever, local swelling, nodules, muscle weakness, muscle pain, and may be accompanied by local skin congestion or no apparent abnormalities. As a result, clinical misdiagnosis and mistreatment are common. It is commonly seen in immunocompromised patients, such as those with HIV/AIDS, diabetes, and connective tissue diseases, as well as patients using steroids, immunosuppressants, and biologics [22]. The main routes of transmission are local tissue spread, hematogenous dissemination, and lymphatic system dissemination. Iatrogenic infections via injection, puncture, or surgical trauma have also been reported [23, 24]. Among the previously reported 21 cases, three patients had abscesses in two different sites who had a long history of steroid use due to different underlying diseases or misdiagnosis. This may have promoted the hematogenous dissemination of *M. tuberculosis*, leading to the occurrence of multiple muscle abscesses.

**Table 1:** Overview of previous cases of extremity tuberculous muscle abscess reported in the literature

Case	Gender	Age (year)	Infection sites	Duration	Underlying diseases	Complicated with PTB	Treatment options	Treatment outcome	References			
1	Female	45	Left thigh	3 moinths	NA	No	HRZE + puncture	Improved	[6]			
2	Male	83	Left lower leg	NA	Addison’s disease & Paget’s disease	No	HRZ	Cured	[7]			
3	Male	45	Right palm	2 weeks	Kidney transplantation	Yes	HRZ	Cured	[8]			
4	Male	0.9	Left thigh	8 months	NA	No	HRZE/HR	Cured	[9]			
5	Female	41	Left forearm	6 months	NA	No	HRZE	Cured	[10]			
6	NA	0.5	Thigh	NA	NA	No	Antituberculosis drugs	Cured	[11]			
7	Male	62	Right thigh	12 months	Total hip arthroplasty	No	Antituberculosis drugs	Cured	[12]			
8	Male	52	Left thigh	6 months	Dermatomyositis	No	HRZE	Cured	[13]			
9	Male	48	Left thigh	2 months	NA	No	HRZ + puncture	Cured	[14]			
10	Male	46	Right upper arm	10 days	Dermatomyositis	No	Isoniazid + rifampin + ethambutol + moxifloxacin	NA	[15]			
11	Male	71	Right thigh	2 months	Lymphatic tuberculosis	No	HRZE + drainage	Cured	[15]			
12	Female	46	Right thigh	1 month	SLE and APS	No	HRZE + drainage	Cured	[15]			
13	Male	11	Right upper arm	2 months	NA	No	HRZE + drainage	Cured	[16]			
14	Male	2	Right lower leg	1.5 months	Upper respiratory tract infection	No	HRZE/HRE	Cured	[17]			
15												
16	Female	21	Right thigh	1 month	NA	No	HRZE/HR	Cured	[18]			
17	Male	47	Left upper arm and left thigh	1 month	Dermatomyositis	No	HRZE + drainage + streptomycin injection	Cured	[19]			
18	Male	25	Back	3 months	NA		HRZE	Cured	[20]			
19	Female	47	Right upper arm	NA	NA	No	HRZE	Cured	[21]			

HRZE, fixed-dose combination with isoniazid(H)-rifampicin(R)-ethambutol(E)-pyrazinamide(Z);SLE, systemic lupus erythematosus; APS, antiphospholipid syndrome; PTB, pulmonary tuberculosis; NA, not applicable

The patient in this case presented with fatigue in the early stages of the disease, progressive swelling in the left lower limb and right upper limb, and a sharp increase in creatine kinase levels to 25,170 U/L. Additionally, the patient exhibited malar rash skin lesions, positive results for multiple rheumatoid immune-related antibodies, and was initially misdiagnosed at a primary hospital with an overlap syndrome of SLE and dermatomyositis. The patient received high-dose steroids and immunosuppressants, potentially causing a considerable increase in M. tuberculosis growth and its spread through the bloodstream to the subcutaneous soft tissue and muscle. This led to a prolonged high fever and systemic toxic symptoms. Generally, the development of muscular TB infection can be divided into three stages: the first stage is characterized by fever, muscle pain, and swelling of the muscle and soft tissue, during which early abscess formation has not yet occurred, making a definitive diagnosis extremely difficult; the second stage is characterized by fever, muscle tenderness, and increased white blood cell count, with the presence of muscle abscesses that can be aspirated locally; the third stage is characterized by systemic TB toxic symptoms and bacteremia, with a mortality rate of up to 10% [25, 26]. During the patient’s visit to the previous hospital, the patient was likely in the first stage, with limb swelling but no obvious signs or symptoms of infection. Upon admission to our hospital, the patient displayed a prolonged high fever, notable soreness in the swollen limb areas, a substantial rise in white blood cell count, and imaging results indicating several signs of soft tissue infection. The patient was currently experiencing the second stage of muscular tuberculosis infection. During the process of establishing a definitive diagnosis, the patient’s condition further worsened, progressing to the third stage, with symptoms of mental fatigue, dizziness, headache, night sweats, and poor appetite, indicating systemic TB toxic symptoms and a highly dangerous condition. Therefore, it is crucial to correctly identify extrapulmonary muscular TB infection in the early stages.

Patients with isolated muscular tuberculosis infection may not exhibit typical symptoms of pulmonary tuberculosis in the initial phases, which complicates the diagnostic process. Diagnosis primarily relies on pathological examination of the affected muscle tissue, culture of *M. tuberculosis*, and PCR identification. Imaging examinations of the muscles can help accurately “locate” and “characterize” the affected muscle groups, guiding the selection of clinical muscle tissue samples and abscess aspiration [27]. For example, local ultrasound examinations often show hypoechoic or irregularly echoic local masses. CT and MRI scans typically reveal well-defined soft tissue mass shadows with lower or equal density compared to normal muscle, but they cannot truly differentiate between muscular TB and other muscular lesions, limiting their diagnostic value. Studies have shown that ^18^F-FDG PET imaging demonstrates moderate radioactive accumulation in the peripheral area and low accumulation in the central caseous necrotic area, which is a specific manifestation of tuberculous abscess formation. However, it is important to note that TB lesions can also take up ^18^F-FDG and may be difficult to distinguish from malignant tumors [28]. Culture of *M. tuberculosis* remains the gold standard for TB detection and drug susceptibility testing, but it is time-consuming and has a high false-negative rate, which does not meet the need for rapid diagnosis. PCR detection methods can quickly and accurately diagnose muscular TB infection, but they have limitations including a high percentage of false-positive results, the requirement for unique primers for different strains and subtypes, and the inability to differentiate between living and dead bacteria. PPD tests and T-SPOT tests have low positivity rates in patients with muscular TB. The technology mNGS is suitable for the diagnosis and differential diagnosis of suspected PTB, extrapulmonary TB, and Non-tuberculous mycobacteria diseases with negative pathogen results. It is also applicable for detecting drug-resistant gene mutations in clinically abundant specimens or strains, as well as for diagnosing drug resistance in *M. tuberculosis*. Compared to culture methods, mNGS has the advantages of high efficiency and rapidity. In a cohort study conducted by Zhou et al. showed that the diagnostic ability of mNGS for extrapulmonary TB is comparable to that of Xpert MTB/RIF [29]. The mNGS assay is more effective in identifying small amounts of M. tuberculosis in clinical samples from extrapulmonary TB cases such tuberculous meningitis, lymph node TB, bone and joint TB, and tuberculous pericarditis. Zhang et al. reported a case of nasopharyngeal carcinoma combined with muscular TB, in which swelling was palpable in the right hip and knee joints. Metagenomic next-generation sequencing suggested the presence of seven sequences from the *M. tuberculosis* complex, with a relative abundance of only 0.02%. Histopathological examination of a biopsy from the right knee lateral soft tissue showed caseous necrosis, and the interferon-gamma release assay was positive (+), leading to a final diagnosis of muscular TB [30]. Therefore, the high sensitivity of mNGS can be observed. According to the consensus of domestic experts, the detection of one specific sequence is sufficient to determine a positive result for clinically significant and difficult-to-detect pathogens such as *M. tuberculosis* [31]. Despite the disadvantages of high cost, specimen and data processing requirements, mNGS has certain advantages in the early, rapid, sensitive, and specific diagnosis of extrapulmonary TB infections in the musculoskeletal system.

The treatment of extrapulmonary TB and PTB is based on the principles of early, regular, comprehensive, appropriate, and combined systemic chemotherapy. Currently, there are no treatment guidelines or expert consensus specifically for muscular TB infections. In this study, 20 cases of tuberculous muscle abscess in the limbs were treated with standardized chemotherapy regimens (including isoniazid, rifampicin, pyrazinamide, ethambutol, etc.) for systemic anti-tuberculosis treatment. The total duration of treatment ranged from 6 to 9 months, which is longer than the treatment duration for initial active PTB. This may be related to factors such as patient age, extent of muscle involvement, size of abscesses, and immune status. Some patients underwent abscess puncture or incision and drainage, and one patient received intracavitary injection of streptomycin, which resulted in rapid abscess absorption [19]. Therefore, in this case, in addition to initiating the standard four-drug chemotherapy regimen, abscess puncture and drainage were performed, followed by intracavitary injection of streptomycin. After one month, the amount of pus drained from the cavity decreased significantly from 300 ml to 50 ml. Follow-up ultrasound examination after four months indicated the disappearance of the cavity, and the patient was able to walk independently without muscle pain or weakness. This suggests that this treatment regimen has a significant therapeutic effect on tuberculous muscle abscesses.

We report a case of SLE complicated by multiple tuberculous muscle abscesses, which is rare in clinical practice. Muscular involvement in extrapulmonary TB is uncommon, and the elevated muscle enzymes can easily be misdiagnosed as dermatomyositis, leading to delayed diagnosis and treatment. The application of mNGS in extrapulmonary TB is becoming increasingly popular, but its use in detecting muscular TB is limited. This case suggests that mNGS can provide an early and rapid diagnosis of muscular TB infection, facilitating the timely use of anti-tuberculosis drugs. In addition to traditional anti-tuberculosis treatment, ultrasound-guided percutaneous aspiration and intracavitary injection of streptomycin for the treatment of tuberculous muscle abscesses are simple, safe, and effective procedures that are worth promoting in clinical practice.

## Data Availability

Data sharing is not applicable to this article as no datasets were generated or analyzed during the current study.
